# Are occupational and environmental noises associated with periodontitis? Evidence from a Korean representative cross-sectional study

**DOI:** 10.1186/s12889-021-10672-5

**Published:** 2021-03-29

**Authors:** Dong-Hun Han, Mi-Sun Kim

**Affiliations:** 1 Department of Preventive and Social Dentistry, Seoul National University School of Dentistry, 1 Gwanak-ro, Gwanak-gu, Seoul, 08826 South Korea; 2 Dental Research Institute, Seoul National University, Seoul, South Korea; 3 Department of Dental Hygiene, Kyungdong University, Wonju, South Korea

**Keywords:** Epidemiology, Noise, Periodontitis, Risk factor

## Abstract

**Background:**

Evidences have shown that noise could be a risk factor for cardiovascular and metabolic diseases. Since periodontitis and CVD are characterized by inflammation, it is reasonable to doubt that occupational/environmental noise is a risk factor for periodontitis. The aim of this study was to examine the relationship between occupational/environmental noise and periodontitis in a nationally representative sample of Korean adults.

**Methods:**

This cross-sectional study used data from the 7th Korean National Health and Nutrition Examination Survey. The study sample included 8327 adults aged 40 to 80 years old. Noise exposure and the duration of the exposure were assessed with self-report questionnaires. The dependent variable was periodontitis. Age, gender, place of residence, income, marital status, smoking, frequency of daily tooth brushing, recent dental checkup, and diabetes were included as covariates. Logistic regression analyses estimated the association between noise exposure and periodontitis.

**Results:**

Those who were exposed to environmental noise during their lifetime had an increased prevalence of severe periodontitis (odds ratio [OR] 1.88; 95% confidence interval [CI] 1.05 to 3.40), and this association was strengthened as the duration of the environmental noise exposure was longer (OR of > 120 months 2.35 and OR of ≤120 months 1.49). There was a combined relationship for severe periodontitis between occupational and environmental noise exposure (OR of both exposures 2.62, OR of occupational exposure only 1.12, and OR of environmental exposure only 1.57).

**Conclusion:**

Our study shows that noise exposure is associated with periodontitis, and the association was higher in the synergism between occupational and environmental interaction.

## Background

As the prevalence of chronic diseases has increased since the late 20th and early twenty-first century, interest in environmental factors for chronic diseases has increased [1]. Especially, noise is becoming increasingly evident in epidemiological studies. Although global estimates of noise exposure are scarce, and methods vary widely, the prevalence of noise exposure at work were 25% among U.S. workers [2], 15% in Canada [3], 20% in the European Union [4], and 20% in Australia [5]. The prevalence of occupational noise exposure in Korea was 20.7% (male 26.5% and female 12.8%) [6]. According to the road traffic noise exposure survey of Korea, 12.6% of the population was exposed to noise during daytime (06:00–22:00) and 52.7% exposed to noise during nighttime (22:00–06:00) [7]. Not only studies have reported that occupational noise exposure is associated with cardiovascular disease (CVD) and deaths [8–11], but within the last decade, a number of studies have been published that address the link between environmental noise (road, aircraft, and railway noise) and CVD [12, 13].

Periodontitis is a well-known chronic inflammatory disease [14]. The prevalence of periodontitis was 23.4% (male 30.9% and female 18.1%) among Koreans adults (19 years old or more). Among the Korean over 30 years old, the prevalence was increased to 29.1% (male 38.4% and female 22.6%). It also showed gap between the lowest income level and the highest (the lowest 27.5% and the highest 20.2%) [15]. Previous studies have shown that those who had periodontitis are prone to dissemination of oral bacteria or endotoxins, which lead to systemic inflammation through an increase in pro-inflammatory cytokines such as tumor necrosis factor-α and interleukin-6 and oxidative stress [16, 17]. On the contrary, the effect of Type 2 Diabetes Mellitus on the inflammatory status of the periodontal tissue is well established. The hyperglycaemia conditions augment the pro-inflammatory response in the periodontal environment [18]. That is, periodontitis and systemic diseases share a common disease-causing factor called inflammation.

Up to date, only one study showed that those who exposed to occupational noise had an increased prevalence of periodontitis (OR = 1.34, 95% CI 1.06 to 1.70) [19], whereas the accumulated evidence on the link of occupational and environmental noise with CVD and the shared common pathophysiology of periodontitis and CVD.

Therefore, this study investigated whether excess noise exposure in and out of the workplace is associated with periodontitis using a representative sample from the 7th Korea National Health and Nutrition Examination Survey (KNHANES VII) 2016–2018. Additionally, hearing protection during occupational noise exposure was also assessed.

## Methods

### Study population

This study used data from the 2016–2018 stage of KNHANES VII, a cross-sectional and nationwide survey performed by the Korea Centers for Disease Control and Prevention (KCDC). KNHANES is a nationwide cross-sectional survey conducted every year, and its target population comprises nationally representative non-institutionalized civilians in Korea. Each survey year includes a new sample of about 10,000 individuals aged 1 year and over. The target population of KNHANES comprises non-institutionalized Korean citizens residing in Korea. The sampling plan follows a multi-stage clustered probability design. For example, 192 primary sampling units (PSUs) were drawn from approximately 200,000 geographically defined PSUs for the whole country. A PSU consisted of an average of 60 households, and 20 final target households were sampled for each PSU using systematic sampling; in the selected households, individuals aged 1 year and over were targeted. All statistics of this survey have been calculated using sample weights assigned to sample participants [20].

Initially, a total of 24,269 individuals (11,071 males and 13,198 females) participated in the KNHANES VII survey. Among them, the noise exposure history was surveyed for those 40 or more years old which were 9450 participants (4054 males and 5396 females), and periodontal health status was measured for 13,789 participants (6119 males and 7670 females). The exclusion criteria consisted of three items: (i) those aged < 40 years, (ii) edentate, and (iii) those missing values in the health assessment or questionnaires. The final sample size for our analysis was 8327 (3572 males and 4755 females). Informed consent was obtained from all participants of the KNHANES. This study was approved by the Institutional Review Board of the Korean Centers for Disease Control and Prevention and was conducted in accordance with the Helsinki Declaration of 1975, as revised in 2013.

### Noise exposure

Assessment of occupational and environmental noise exposure was obtained from two types of self-report questionnaires. The first question for occupational noise exposure was “Have you been exposed to loud noise for more than three months during work? A loud noise means that you have to raise your voices for a conversation and refers to machines or generators.” The second question for environmental noise exposure was “Have you been exposed to loud noise for more than five hours per week except at work? A loud noise means that you have to raise your voices to keep a conversation and refers to a car, truck, motorcycle, machine, or loud music.” Those who answered yes to the occupational or environmental noise exposure question were asked to answer the duration of the loud noise exposure by months. Then, the duration of noise exposure was divided into three groups based on the median 120 months (no exposed, ≤120 months, and > 120 months). Additionally, those who answered yes to the occupational noise exposure question were asked “Did you wear hearing protection devices during work?”

### Sociodemographic and health related variables

All participants were asked about sociodemographic and health behaviors variables by trained interviewers. Age was asked by continuous (years). Gender, place of residence and marital status were categorized into two groups: male vs. female for gender, urban vs. rural for place of residence, and married vs. single for marital status. Income was measured by monthly household income as a continuous variable. Then it was calculated into an equivalised monthly household income ([monthly overall household income]/[household size]^-0.5^). Income was categorized into four groups: from lowest quartile to highest quartile for income. Occupation was classified into three groups: white collars (manager, professionals, and clerical workers), blue collars (Skilled agricultural, forestry and fishery workers, Plant and machine operators and assemblers, and elementary occupations), and pink collars (service and sales workers). Health behavior variables were categorized into two groups: current vs. non-current smoker for smoking and no vs. yes for daily toothbrushing habit and recent dental checkup.

A blood sample was obtained from the antecubital vein of each participant after fasting for more than 8 h to measure the concentration of serum fasting plasma glucose with an Automatic Analyzer 7600 (Hitachi, Tokyo, Japan) using kits (Daiichi, Tokyo, Japan) [21]. Physicians classified diabetes status as follows: no diabetes (normal fasting plasma glucose level, < 100 mg/dl), pre-diabetes (< 126 mg/dl), and diabetes (≥126 mg/dl or anti-diabetic medication use).

### Periodontal health assessment

Dentists conducted an oral health examination in the survey. The World Health Organization (WHO) community periodontal index (CPI) was used to assess periodontitis [22]. Periodontitis was defined as a CPI greater than or equal to “code 3”, which is given to more than one sextant that harbors 4–5 mm deep or deeper pockets in the index teeth. Severe periodontitis was defined as a CPI equal to “code 4”, which is given to more than one sextant that harbors pockets 6 mm deep or deeper in the index teeth. The index teeth numbers were 11, 16, 17, 26, 27, 31, 36, 37, 46, and 47, respectively.

### Statistical analyses

The characteristics of the study subjects by periodontitis and severe periodontitis were presented with frequency distributions for the categorical variables and means for the continuous variables. Complex samples chi-square tests for the categorical variables and complex samples independent t-tests/ANOVA for the continuous variables were used to assess the associations of the sociodemographic and health related factors with periodontal health status and noise exposure. The occupational and environmental noise exposure, duration of noise exposure, hearing protection, and the interaction between occupational and environmental noise exposure according to periodontitis were also examined.

Adjusted odds ratio (OR_*adj*_) of periodontitis and severe periodontitis with noise exposure adjusting for confounders were obtained by complex samples logistic regression. Model 1 was adjusted for age and gender, model 2 was controlling for age, gender, place of residence and income, and model 3 was adjusted for age, gender, place of residence, income, occupation, marital status, diabetes, smoking, toothbrushing, and recent dental checkup. IBM SPSS Statistical Software (version 22.0, IBM Corp, Armonk, NY, 2013) was used for all analysis. All reported *P* values are 2-tailed. P values ≤.05 were considered as statistically significant.

## Results

Table 1 presents the descriptive summary of the socio-demographic and health related characteristics according to periodontitis and mild & severe periodontitis. Among the 8327 participants in the sample, 3339 (40.1%) had periodontitis and 891 (10.7%) had severe periodontitis. The age of the periodontitis group had higher means compared to no periodontitis group. Those with the following socio-demographic and health related characteristics showed more periodontitis and severe periodontitis: male, rural residency, the lowest income, blue collar, those who had diabetes, current smokers, toothbrushing habit and recent dental checkup.

**Table 1:** Characteristics of population by periodontal disease

Variables	Periodontitis		*P*	Mild & severe periodontitis			*P*
	No (*n* = 4988), n (%)	Yes (*n* = 3339), n (%)		No (*n* = 4988), n (%)	Mild (*n* = 2448), n (%)	Severe (*n* = 891), n (%)	
Age, mean (95% confidence interval) (years)	56.74 (56.41–57.06)	60.89 (60.52–61.26)	< 0.001^*^	56.74 (56.41–57.06)^a^	60.99 (60.55–61.43)^b^	60.62 (59.91–61.34)^b^	< 0.001^§^
Gender							
Male (*n* = 3572)	1805 (34.1)	1767 (51.5)	< 0.001	1805 (34.1)	1222 (49.0)	545 (58.5)	< 0.001
Female (*n* = 4755)	3183 (65.9)	1572 (48.5)		3183 (65.9)	1226 (51.0)	346 (41.5)	
Residence							
Urban (*n* = 6706)	4165 (85.8)	2541 (77.9)	< 0.001	4165 (85.8)	1882 (79.2)	659 (74.3)	< 0.001
Rural (*n* = 1621)	823 (14.2)	798 (22.1)		823 (14.1)	566 (20.8)	232 (25.7)	
Income							
I (lowest) (*n* = 2024)	1127 (22.2)	897 (27.1)	< 0.001	1127 (22.2)	645 (26.1)	252 (29.8)	< 0.001
II (*n* = 2103)	1228 (24.0)	875 (25.8)		1228 (24.0)	632 (24.8)	243 (28.3)	
III (*n* = 2098)	1280 (26.0)	818 (24.3)		1280 (26.0)	604 (24.8)	214 (23.0)	
IV (highest) (*n* = 2102)	1353 (27.8)	749 (22.8)		1353 (27.8)	567 (24.2)	182 (19.0)	
Occupation							
White collar (*n* = 1662)	1160 (22.3)	502 (14.4)	< 0.001	1160 (22.3)	371 (14.9)	131 (13.2)	< 0.001
Blue collar (*n* = 2156)	1104 (21.1)	1052 (30.3)		1104 (21.1)	743 (29.3)	309 (32.8)	
Pink collar (*n* = 4509)	2724 (56.6)	1785 (55.3)		2724 (56.6)	1334 (55.8)	451 (54.0)	
Marital status							
Married (*n* = 8018)	4794 (96.5)	3224 (96.7)	0.712	4794 (96.5)	2369 (97.0)	855 (95.9)	0.402
Single (*n* = 309)	194 (3.5)	115 (3.3)		194 (3.5)	79 (3.0)	36 (4.1)	
Diabetes							
No (*n* = 4593)	3011 (60.7)	1582 (49.1)	< 0.001	3011 (60.7)	1196 (50.4)	386 (45.3)	< 0.001
Prediabetes (*n* = 2367)	1338 (27.2)	1029 (29.7)		1338 (27.2)	757 (29.9)	272 (29.1)	
Diabetes (*n* = 1367)	639 (12.1)	728 (21.2)		639 (12.1)	495 (19.7)	233 (25.6)	
Smoking							
Quit/never smoker (*n* = 6989)	4411 (89.0)	2578 (77.9)	< 0.001	4411 (89.0)	1927 (79.1)	651 (74.7)	< 0.001
Current smoker (*n* = 1338)	577 (11.0)	761 (22.1)		577 (11.0)	521 (20.9)	240 (25.3)	
Toothbrushing							
No (*n* = 121)	60 (1.1)	61 (1.8)	0.023	60 (1.1)	45 (1.8)	15 (1.9)	0.266
Yes (*n* = 8206)	4928 (98.9)	3278 (98.2)		4928 (98.9)	2402 (98.1)	876 (98.1)	
Recent dental checkups							
No (*n* = 5230)	2950 (58.4)	2280 (68.4)	< 0.001	2950 (58.4)	1680 (68.6)	600 (67.8)	< 0.001
Yes (*n* = 3097)	2038 (41.6)	1059 (31.6)		2038 (41.6)	768 (31.4)	291 (32.2)	

P values were obtained by complex samples chi-square test

^*^ *P* value was obtained by complex samples independent t-test

^§^ *P* value was obtained by complex samples ANOVA

The characteristics of participants according to occupational and environmental noise exposure were shown in Table 2. The prevalence of occupational noise exposure was 15.5% and environmental noise exposure was 2.1%. Those who were exposed to occupational noise were younger than those who were not exposed to occupational noise. Male, rural residency, the lowest income, blue collar, single, those who had prediabetes & diabetes, and current smokers had more occupational noise exposure. In case of environmental noise exposure, the lower income, single, those who had diabetes, and current smokers were more exposed.

**Table 2:** Characteristics of population by occupational and environmental noise

Variables	Occupational noise		*P*	Environmental noise		*P*
	No (*n* = 7033), n (%)	Yes (*n* = 1294), n (%)		No (*n* = 8150), n (%)	Yes (*n* = 177), n (%)	
Age, mean (95% confidence interval) (years)	58.57 (58.30–58.84)	57.49 (56.91–58.07)	0.001^*^	58.41 (58.15–58.66)	58.24 (56.61–59.88)	0.853^*^
Gender						
Male (*n* = 3572)	2784 (37.8)	788 (58.4)	< 0.001	3498 (40.1)	74 (41.8)	0.905
Female (*n* = 4755)	4249 (62.2)	506 (41.6)		4652 (57.9)	103 (58.2)	
Residence						
Urban (*n* = 6706)	5690 (83.1)	1016 (80.2)	0.184	6572 (80.6)	134 (75.7)	0.102
Rural (*n* = 1621)	1343 (16.9)	278 (19.8)		1578 (19.4)	43 (24.3)	
Income						
I (lowest) (*n* = 2024)	1639 (23.1)	385 (29.9)	< 0.001	1967 (24.1)	57 (32.2)	0.005
II (*n* = 2103)	1748 (24.3)	355 (26.8)		2045 (25.1)	58 (32.8)	
III (*n* = 2098)	1799 (25.6)	299 (24.1)		2068 (25.4)	30 (16.9)	
IV (highest) (*n* = 2102)	1847 (27.0)	255 (19.2)		2070 (25.4)	32 (18.1)	
Occupation						
White collar (*n* = 1662)	1521 (20.8)	141 (10.4)	< 0.001	1639 (20.1)	23 (13.0)	0.195
Blue collar (*n* = 2156)	1590 (21.6)	566 (41.8)		2104 (25.8)	52 (29.4)	
Pink collar (*n* = 4509)	3922 (57.6)	587 (47.8)		4407 (54.1)	102 (57.6)	
Marital status						
Married (*n* = 8018)	6785 (96.8)	1233 (95.5)	0.027	7855 (96.4)	163 (92.1)	0.039
Single (*n* = 309)	248 (3.2)	61 (4.5)		295 (3.6)	14 (7.9)	
Diabetes						
No (*n* = 4593)	3926 (56.6)	667 (53.4)	0.136	4507 (55.3)	86 (48.6)	0.037
Prediabetes (*n* = 2367)	1971 (28.0)	396 (29.3)		2317 (28.4)	50 (28.2)	
Diabetes (*n* = 1367)	1136 (15.4)	231 (17.3)		1326 (16.3)	41 (23.2)	
Smoking						
Quit/never smoker (*n* = 6989)	6025 (86.4)	964 (75.0)	< 0.001	6850 (84.0)	139 (78.5)	0.004
Current smoker (*n* = 1338)	1008 (13.6)	330 (25.0)		1300 (16.0)	38 (21.5)	
Toothbrushing						
No (*n* = 121)	102 (1.5)	19 (1.5)	0.876	117 (1.4)	4 (2.3)	0.975
Yes (*n* = 8206)	6931 (98.5)	1275 (98.5)		8033 (98.6)	173 (97.7)	
Recent dental checkups						
No (*n* = 5230)	4413 (62.1)	817 (63.4)	0.465	5118 (62.8)	112 (63.3)	0.539
Yes (*n* = 3097)	2620 (37.9)	477 (36.6)		3032 (37.2)	65 (36.7)	

*P* values were obtained by complex samples chi-square test

^*^ *P* value was obtained by complex samples independent t-test

The various noise exposures were associated with periodontitis and severe periodontitis (Table 3); 17.8% and 20.1% of the periodontitis and severe periodontitis participants had been exposed to occupational noise while 13.6% of the no periodontitis had been exposed. There was a greater number of periodontitis and severe periodontitis participants among those who exposed more than 120 months. However, the exposure and duration of the environmental noise were associated only with severe periodontitis. The interaction of occupational and environmental noise exposure was associated with periodontitis and severe periodontitis. Especially, those who were exposed to both occupational and environmental noise exhibited a higher prevalence of severe periodontitis.

**Table 3:** Crude association between noise exposure and periodontitis

Variables	Periodontitis		*P*	Mild & severe periodontitis			*P*
	No (*n* = 4988), n (%)	Yes (*n* = 3339), n (%)		No (*n* = 4988), n (%)	Mild (*n* = 2448), n (%)	Severe (*n* = 891), n (%)	
Occupational noise exposure							
No (*n* = 7033)	4307 (86.4)	2726 (82.2)	< 0.001	4307 (86.4)	2023 (83.0)	703 (79.9)	< 0.001
Yes (*n* = 1294)	681 (13.6)	613 (17.8)		681 (13.6)	425 (17.0)	188 (20.1)	
Duration of occupational noise exposure							
No (*n* = 7033)	4307 (86.4)	2726 (82.2)	< 0.001	4307 (86.4)	2023 (79.9)	703 (79.9)	< 0.001
≤ 120 months (*n* = 793)	443 (8.8)	350 (9.9)		443 (8.8)	244 (9.7)	106 (10.6)	
> 120 months (*n* = 501)	238 (4.8)	263 (7.9)		238 (4.8)	181 (7.4)	82 (9.4)	
Hearing protection in workplace							
No exposure (*n* = 7033)	4307 (86.4)	2726 (82.2)	< 0.001	4307 (86.4)	2023 (83.0)	703 (78.9)	< 0.001
Occupational exposure with protection (*n* = 308)	164 (3.3)	144 (3.9)		164 (3.3)	89 (3.3)	55 (5.5)	
Occupational exposure without protection (*n* = 986)	517 (10.3)	469 (13.9)		517 (10.3)	336 (13.7)	133 (14.6)	
Environmental noise exposure							
No (*n* = 8150)	4894 (98.3)	3256 (97.6)	0.060	4894 (98.3)	2399 (98.1)	857 (96.4)	0.001
Yes (*n* = 177)	94 (1.7)	83 (2.4)		94 (1.7)	49 (1.9)	34 (3.6)	
Duration of environmental noise exposure							
No (*n* = 8150)	4894 (98.3)	3256 (97.6)	0.137	4894 (98.1)	2399 (98.0)	857 (96.2)	0.004
≤ 120 months (*n* = 90)	48 (0.9)	42 (1.2)		48 (1.0)	22 (0.9)	19 (2.1)	
> 120 months (*n* = 87)	46 (0.8)	41 (1.2)		46 (0.9)	27 (1.1)	15 (1.7)	
Interaction between occupational & environmental noise							
Both no exposure (*n* = 6913)	4241 (85.2)	2672 (80.7)	< 0.001	4241 (85.2)	1988 (81.6)	684 (78.0)	< 0.001
Only environmental noise exposure (*n* = 120)	66 (1.2)	54 (1.5)		66 (1.2)	35 (1.4)	19 (1.9)	
Only occupational noise exposure (*n* = 1237)	653 (13.1)	584 (17.0)		653 (13.1)	411 (16.5)	173 (18.4)	
Both exposure (*n* = 57)	28 (0.5)	29 (0.8)		28 (0.5)	14 (0.5)	15 (1.7)	

*P* values were obtained by complex samples chi-square test

In Table 4, those who had been exposed to occupational noise were more likely to have periodontitis (odds ratio [OR] = 1.25) and severe periodontitis (OR = 1.36) in model 1. When exposed to occupational noise more than 120 months, the prevalence of periodontitis (OR = 1.20 and 1.33 for ≤120 months and > 120 months, respectively) and severe periodontitis (OR = 1.29 and 1.48 for ≤120 months and > 120 months, respectively) were higher in model 1. However, these associations were disappeared according to the serial adjustments (model 2 and model 3). Environmental noise exposure was associated with periodontitis (OR = 1.43) and severe periodontitis (OR = 2.30), and when the exposures was more than 120 months, the risk of severe periodontitis was higher (OR = 1.78 and 2.74 for ≤120 months and > 120 months, respectively) in model 1. These associations between environmental noise and severe periodontitis were persisted even after controlling for socioeconomic and health related factors (model 2 and model 3). There was a combined effect between occupational and environmental noise exposure. The OR of both occupational and environmental noise exposure for severe periodontitis (OR = 3.52) was greater than the ORs of occupational and environmental noise exposure in model 1. The associations were attenuated but still significant when controlling for socioeconomic and health related factors (model 2 and model 3).

**Table 4:** Association between noise exposure and periodontitis

Noise exposure	Dependent: periodontitis (reference = no periodontitis)			Dependent: severe periodontitis (reference = no periodontitis)		
	Model 1	Model 2	Model 3	Model 1	Model 2	Model 3
Occupational noise exposure						
No	1.00 (reference)	1.00 (reference)	1.00 (reference)	1.00 (reference)	1.00 (reference)	1.00 (reference)
Yes	**1.25 (1.06–1.48)**	**1.20 (1.02–1.42)**	1.11 (0.94–1.32)	**1.36 (1.08–1.72)**	**1.28 (1.02–1.62)**	1.17 (0.92–1.48)
Duration of occupational noise exposure						
No	1.00 (reference)	1.00 (reference)	1.00 (reference)	1.00 (reference)	1.00 (reference)	1.00 (reference)
≤ 120 months	1.20 (0.98–1.48)	1.14 (0.93–1.39)	1.07 (0.87–1.31)	1.29 (0.96–1.72)	1.18 (0.88–1.58)	1.07 (0.80–1.44)
> 120 months	**1.33 (1.04–1.70)**	**1.31 (1.03–1.67)**	1.19 (0.93–1.51)	**1.48 (1.08–2.03)**	**1.43 (1.04–1.97)**	1.31 (0.95–1.80)
Hearing protection in workplace						
No exposure	1.00 (reference)	1.00 (reference)	1.00 (reference)	1.00 (reference)	1.00 (reference)	1.00 (reference)
Occupational exposure with protection	1.12 (0.85–1.48)	1.07 (0.81–1.41)	1.00 (0.75–1.33)	1.49 (0.98–2.26)	1.40 (0.91–2.14)	1.27 (0.83–1.95)
Occupational exposure without protection	**1.29 (1.07–1.56)**	**1.24 (1.04–1.49)**	1.15 (0.95–1.38)	**1.33 (1.02–1.71)**	1.24 (0.96–1.60)	1.13 (0.88–1.46)
Environmental noise exposure						
No	1.00 (reference)	1.00 (reference)	1.00 (reference)	1.00 (reference)	1.00 (reference)	1.00 (reference)
Yes	**1.43 (1.01–2.02)**	1.34 (0.93–1.93)	1.21 (0.82–1.76)	**2.30 (1.39–3.78)**	**2.10 (1.38–3.18)**	**1.88 (1.05–3.40)**
Duration of environmental noise exposure						
No	1.00 (reference)	1.00 (reference)	1.00 (reference)	1.00 (reference)	1.00 (reference)	1.00 (reference)
≤ 120 months	1.25 (0.81–1.93)	1.17 (0.75–1.83)	1.10 (0.68–1.77)	1.78 (0.97–3.24)	1.62 (0.74–3.57)	1.49 (0.64–3.48)
> 120 months	1.65 (0.98–2.79)	1.55 (0.90–2.65)	1.33 (0.78–2.25)	**2.74 (1.57–4.78)**	**2.69 (1.40–5.20)**	**2.35 (1.18–4.68)**
Interaction between occupational & environmental noise						
Both no exposure	1.00 (reference)	1.00 (reference)	1.00 (reference)	1.00 (reference)	1.00 (reference)	1.00 (reference)
Only environmental noise exposure	1.42 (0.94–2.17)	1.35 (0.89–2.05)	1.23 (0.81–1.86)	1.86 (1.00–3.50)	1.74 (0.90–3.37)	1.57 (0.80–3.08)
Only occupational noise exposure	**1.25 (1.05–1.48)**	**1.20 (1.01–1.42)**	1.11 (0.93–1.32)	**1.30 (1.02–1.66)**	1.23 (0.97–1.56)	1.12 (0.88–1.43)
Both exposure	1.60 (0.86–3.00)	1.45 (0.75–2.82)	1.23 (0.61–2.48)	**3.52 (1.63–7.63)**	**3.06 (1.30–7.22)**	**2.62 (1.03–6.67)**

Model 1 was adjusted for age and gender

Model 2 was adjusted for age, gender, residence, and income

Model 3 was adjusted for age, gender, residence, income, occupation, marital status, diabetes, smoking, toothbrushing, and recent dental checkups,

## Discussion

The aim of this study was to evaluate associations of occupational and environmental noise exposure with periodontal health status among Korean adults aged 40 or more years. According to our results, the severe periodontitis was associated with environmental noise exposure, and the association showed a dose-response relationship as the duration of the noise exposure grew. Although some studies have reported a relationship between occupational noise and health problems [8–11], and other studies also have showed an association between environmental noise and health problems [12, 13], this study is the first to show an association between environmental noise exposure and periodontitis.

In a previous study [19], we found that there was an association between occupational noise exposure and periodontitis. At that time, the noise exposure was measured for currently working workers by a cross-sectional survey, so it was difficult to identify the link between periodontitis which had cumulative characteristics and past occupational noise exposure. Additionally, there was no question about the exposure to environmental noise and duration of the noise exposure. Therefore, the need for further study was raised because a recently conducted KNHANES VII included not only the environmental noise exposure, but also the duration of occupational and environmental noise exposure.

In our study, occupational noise exposure showed similar relationships between periodontitis and severe periodontitis. When the duration of the occupational noise exposure was more than 120 months, increased ORs for periodontitis and severe periodontitis was seen. Results from previous studies on the relationship between occupational noise exposure and CVD reported that when the duration of the occupational noise exposure was longer, the associations were stronger [10, 23–27]. In addition, it is difficult to interpret the results about the opposite ORs for wearing hearing protection devices. Those who did not wear a hearing protection device during work showed a slightly higher OR of periodontitis, while those who did had a much higher OR of severe periodontitis. Although it was hypothesized that the risk of periodontitis would increase if hearing protection devices were not worn despite the exposure to occupational noise, the risk of severe periodontitis increased when hearing protection devices were worn. It can be inferred that people with high levels of occupational noise and a longer duration of exposure wore more hearing protection devices, thereby increasing the risk of severe periodontitis. However, further well-designed studies will be needed to prove this inference. On the other hand, environmental noise exposure showed no association with periodontitis, but a strong association and positive-response relationship with severe periodontitis. With growing interest in the health impacts of environmental noise, the World Health Organization recently published guidelines on environmental noise [28] and studies on the negative health effects of environmental noise have been reported [29, 30]. This study is an exploratory study that adds evidence for the link between environmental noise and periodontal disease to these recent studies.

As a result of our study, exposure to occupational and environmental noise at the same time showed higher association with periodontal disease than exposure to occupational noise and environmental noise, alone. Therefore, there is a need for an innovative public health program that can intervene in vulnerable groups that are simultaneously exposed to occupational and environmental noise.

Although this study found an association between occupational/environmental noise and periodontitis from a representative national Korean sample, some limitations should be noted. First, because the noise exposures of individuals were obtained by recall and the participants may not have recalled their past noise exposure correctly, the results of our study could be biased. The self-reported assessment of noise exposure has been used because of its low expense, logistical ease, and the ability to assess exposures during periods where the worker is inaccessible for direct measurements. Although studies in specific industries have shown good agreement between measured and worker-reported noise levels [31, 32], self-reported survey item performance should have been validated against objective measurements of noise. No information about the severity of noise is another limitation of this study. Especially, the occupational noise exposure should have been measured for hours/day. Second, the cross-sectional design prohibited us from inferring causal relationships. Third, it was necessary to do objective measurements, not subjective surveys, of noise measurements in the workplaces and living environments of the participants. For these reasons, the results of this study should be interpreted with caution.

However, there are several strengths in our study. The data used in this study were a nationally representative sample, and the association between noise and periodontitis was evaluated using multivariate logistic regression analyses after adjusting for confounding factors. This study, thus, can be considered representative and reliable.

## Conclusion

Collectively, there was an association between noise and periodontitis by multivariate logistic regression analyses after adjusting for confounding factors among Korean adults. Noise may be a risk factor for periodontal disease, and the risk of periodontal disease may increase as noise exposure accumulates, so it should be tracked with interest in the oral health effects of noise.

## Data Availability

The datasets generated and/or analyzed during the current study are available in the Korean Centers for Disease Control and Prevention repository, https://knhanes.cdc.go.kr/knhanes/main.do.
